# Arabidopsis Tetraspanins Facilitate Virus Infection *via* Membrane-Recognition GCCK/RP Motif and Cysteine Residues

**DOI:** 10.3389/fpls.2022.805633

**Published:** 2022-03-03

**Authors:** Tingyu Zhu, Yanbiao Sun, Xu Chen

**Affiliations:** ^1^College of Life Sciences, Fujian Agriculture and Forestry University, Fuzhou, China; ^2^Horticultural Plant Biology and Metabolomics Center, Haixia Institute of Science and Technology, Fujian Agriculture and Forestry University, Fuzhou, China

**Keywords:** tetraspanin-3, GCCK/RP motif, cysteines residues, virus infection, distribution

## Abstract

Tetraspanins (TETs) function as key molecular scaffolds for surface signal recognition and transduction *via* the assembly of tetraspanin-enriched microdomains. TETs’ function in mammalian has been intensively investigated for the organization of multimolecular membrane complexes, regulation of cell migration and cellular adhesion, whereas plant TET studies lag far behind. Animal and plant TETs share similar topologies, despite the hallmark of “CCG” in the large extracellular loop of animal TETs, plant TETs contain a plant specific GCCK/RP motif and more conserved cysteine residues. Here, we showed that the GCCK/RP motif is responsible for TET protein association with the plasma membrane. Moreover, the conserved cysteine residues located within or neighboring the GCCK/RP motif are both crucial for TET anchoring to membrane. During virus infection, the intact TET3 protein enhanced but GCCK/RP motif or cysteine residues-deficient TET3 variants abolished the cell-to-cell movement capability of virus. This study provides cellular evidence that the GCCK/RP motif and the conserved cysteine residues are the primary determinants for the distribution and function of TET proteins in Arabidopsis.

## Introduction

The plasma membrane (PM) is a permeable membrane system, which provides a platform of signal activation for pathogen entry or defense to pathogens (Nathalie and Bouhidel, 2014; Heinlein, 2015). Viruses take advantage of cellular membrane to infect host cells in various ways (Burckhardt and Greber, 2009). For instance, clathrin-mediated endocytosis is the major cellular entry pathway for the enveloped virus fusion with PM (Marsh and Helenius, 2006; Miyauchi et al., 2009; Mudhakir and Harashima, 2009). However, PM is not a homogeneous sheet with associated proteins and lipids (Jacobson et al., 2019). The membrane lipid rafts are consisted of liquid-ordered membrane nanoscale domains (<200 nm), which segregated the membrane into a more tightly packed, liquid-ordered phase, and a less tightly packed liquid-disordered phase (Sezgin et al., 2017). Our previous study has shown that salicylic acid (SA) promotes the compartmentalization of membrane nanodomains and increases the proportion of ordered lipid phase, whose organization requires modulation of the lipid nanodomain-specified remorin proteins (Huang et al., 2019).

Besides remorin-associated nanodomains, TET proteins that interact with each other and with diverse membrane-associated proteins establish another type of membrane nanodomains, termed tetraspanin enriched microdomains (TEMs). TETs are a class of highly evolutionarily conserved integral membrane proteins that have been intensively studied in mammals, insects, and fungi (Maecker et al., 1997; Todres et al., 2000; DeSalle et al., 2010). In animals, multiple viruses trigger host cell-cell fusion in a TET-dependent manner, leading to giant-cell or syncytia formation, thereby promoting virus spread (Martin et al., 2005; Hassuna et al., 2009; Jimenez-Jimenez et al., 2019). Animal TETs maintain an interactive network with other membrane proteins, such as integrins and membrane receptors, to organize multi-molecular signaling platforms (Zöller, 2009). In fungi, the tetraspanin-like protein (PLS1) is essential for appressorium-mediated penetration of the fungal pathogen into host plants (Veneault-Fourrey et al., 2005, 2006; Lambou et al., 2008). During plant infection with the pathogens, since peg formation and penetration require the reestablishment of cell polarity at a focal point localized at the base of the appressorium, Pls1 plays a role in the correct localization of the emergence site of the penetration peg (Veneault-Fourrey et al., 2005). Therefore, TETs serve as master organizers integrating within membranes to control the distribution of associated partners, participating in cell adhesion, migration, and cell proliferation (Berditchevski and Odintsova, 1999; Hemler, 2005).

With regard to TETs’ structure, animal and plant TETs share similar topology including four transmembrane (TM) domains (TM1-4), two unequal-size extracellular loops (EC1: small extracellular loop; EC2/LEL: large extracellular loop) with conserved amino acid residues and motifs, small intracellular loop (ICL) and carboxy terminal tails (Hemler, 2005; Kovalenko et al., 2005). Moreover, they seem to share a common ancestor as the conserved intron position of plant TETs appears to be the most ancient intron that presents in animal TETs (Garcia-España and DeSalle, 2009). However, plant TETs are completely different from their metazoan counterparts at the amino acid level, thus failing to identify direct orthologs. Besides, they lack several highly conserved residues in the TM2- ICL-TM3 of the so-called “tetraspanin signature” defined in metazoan TETs (Seigneuret et al., 2001; Kovalenko et al., 2005). In metazoans, the extracellular loop EC2 is consisted of a conserved and a variable subdomain, which are possibly responsible for homodimerization and partner binding of TETs, respectively (Seigneuret et al., 2001; Yánez-Mó et al., 2001; Stipp et al., 2003; Seigneuret, 2006). Hence, EC2 contributes essentially to a specific function of different TETs (Kitadokoro et al., 2001; Stipp et al., 2003). Besides the conserved domains, plant TETs harbor a specific “GCCK/RP” motif, differing in position and sequence from the “CCG” motif present in animal TETs (Wang et al., 2012), implying the specific function of plant TETs. Moreover, a series of conserved cysteine residues in TETs are required for the formation of disulfide bridges and the assembly of TEMs (Hassuna et al., 2009; Zöller, 2009). Compared to animal TETs, plant TETs have nine, rather than four, six, or eight, completely conserved Cys residues (Reimann et al., 2017). The above differences between animal and plant TETs make the possibility that plant specific “GCCK/RP” motif and conserved Cys residues may play a role in specifying plant TET function.

However, in contrast to the considerable advances of animal TETs in the regulation of virus infection (Martin et al., 2005; Hassuna et al., 2009), the functionality of plant TETs are still unclear. 17 *Arabidopsis thaliana* TETRASPANIN (AtTET1-17) genes were found in *Arabidopsis thaliana*, whereas most of them are functional unknown (Cnops et al., 2006). The spatial expression pattern of Arabidopsis TETs has been described in reproductive tissues, suggesting that plant TETs may play roles in intercellular communication (Boavida et al., 2013). So far, only limited TET members have been functionally characterized in plants. The best characterized TET in plants is AtTET1 [also called TORNADO2 (TRN2) and EKEKO]. Genetic data analysis indicates that AtTET1 functions synergistically with TORNADO1 (TRN1), a leucine rich-repeat protein (Cnops et al., 2000, 2006). All *trn* mutants had struck phenotypes: severely dwarfed, with twisted and malformed organs, and sterile (Cnops et al., 2006). Besides, the defective transport and distribution of the plant hormone auxin in *tet1*/*trn2* mutants severely affect leaf symmetry, venation patterning, and root epidermal patterning (Cnops et al., 2006). AtTET1 also determinate the cell fate in the peripheral zone of shoot apical meristem, and the knock-out *Attet1* mutant was sterile due to a defect in megasporogenesis (Chiu et al., 2007; Lieber et al., 2011). However, no obvious phenotype was observed in single *Attet5* and *Attet6* mutants (Wang et al., 2015). *Attet5 Attet6* double mutants display enlarged leaf size due to an increased cell number, increased fresh weight, and longer primary roots, suggesting that AtTET5 and 6 function redundantly in inhibiting cell proliferation during root and leaf growth (Wang et al., 2015). AtTET8 and AtTET9 contribute to exosome formation during fungal infection (Ferrari et al., 2007; Boavida et al., 2013; Cai et al., 2018). TET13 functions related to auxin and lateral root founder cells formation in pericycle (Wang et al., 2015). Taken together, plant TETs might be involved in diverse aspects of plant development, whereas the regulatory mechanisms are still far beyond our understanding.

In this study, we investigated the key motifs and amino acids that determine the subcellular distribution of TET proteins. We found that the PM association of TETs was dependent on the transmembrane domains and extracellular loops. Moreover, we showed that the plant specific “GCCK/RP” motif and several conserved cysteine residues are sufficient for TETs targeting the PM. The membrane association property of TET determines its function in promoting cell-to-cell virus movement. Our findings unraveled the structural specificity of Arabidopsis TET proteins for their specific localization and contribution to virus spread.

## Materials and Methods

### Plant Material and Growth Conditions

Surface-sterilized seeds of *A. thaliana* were sown on growth medium (1 × Murashige and Skoog (MS) mineral salts (Sangon Biotech, Shanghai, China), agar (Bio Basic Inc., Markham, ON, Canada) at 0.8% (w/v), and sucrose (Bio Basic Inc., Markham, ON, Canada) at 1% (w/v), adjusted to pH 5.7. Seeds were stratified at 4°C for 48 h in the dark, then they were grown at the growth chamber at 22°C under 16 h light/8 h dark period with a continuous white light illumination of approximately 100 μmol/m^2^/s m^–2^.

### Vector Construction

All polymerase chain reaction (PCR) amplifications were performed with the primers which were listed in Supplementary Table 1. PrimeSTAR^®^ GXL DNA Polymerase (TaKaRa, Maebashi, Japan) were used for cDNA or genomic DNA amplifications. The resultant fragments were cloned into the entry or destination vectors *via* Gateway technology (Invitrogen, Carlsbad, CA, United States) (listed in Supplementary Table 1). The resultant plasmids were transformed into *Agrobacterium tumefaciens* strain GV3101 for the further Arabidopsis (in *Col-0* ecotype) transformation.

### Plasmid Mutagenesis

For the generation of truncated TET proteins, the upstream and downstream flanking sequences of the designed TET gene were amplified by PCR from the full-length CDS. Then, the resultant DNA fragments were connected by two-step overlapping PCR and further cloned into the destination vector *via* Gateway^®^ cloning technology.^1^

For the generation of site-directed TET mutagenesis, oligonucleotide primers (Supplementary Table 1) containing the single Cysteine (Cys) to Alanine (Ala) were used to introduce mutagenesis by Gibson method. The resultant PCR products were further cloned into destination vector by Gateway cloning. All strategies of vectors and cloning used in this study are listed in Supplementary Table 2.

### Bioinformatics Analysis

The sequences of Arabidopsis TETs were derived from The Arabidopsis Information Resource^2^ and the 2D structure was constructed by UniProt^3^ and PROTTER,^4^ respectively. Phylogenetic tree was generated by using Molecular Evolutionary Genetic Analysis (MEGA) v6.0 (Kumar et al., 2018) software with neighbor-joining (NJ) algorithm, which was verifying by maximum likelihood (ML) method. 1,000 bootstrap replicates were performed to test the significance of nodes. Palmitoylation sites were predicted using Palmitoylation CSS-Palm 4.0 (Ren et al., 2008). Motif1--3 were predicted using MEME Suite.^5^

### Subcellular Localization Assay

For plasmolysis assay, the transformed Arabidopsis root cells were treated with 30% sucrose. For subcellular localization assays, *A. tumefaciens* GV3101 strains (OD_600_ = 0.6) containing the established constructs were infiltrated into 4-week-old *Nicotiana benthamiana* (tobacco) leaves. For co-infiltration, the constructs, ER-tagged mCherry (HDEL) and P19 strains were mixed in the ratio of 1:1:2 (v/v/v). The infiltrated plants were grown in a growth chamber with 16 h light/8 h dark period at 25°C. Two days post-infiltration, the fluorescence signal of tobacco leaves was visualized.

For propidium iodide (PI) staining, detached tobacco leaves were submerged in 30 μM PI (Molecular Probes) at room temperature for 2 min. Leaves were observed using a fluorescence microscope with 535 nm excitation and 615 nm emission filters. For visualization of GFP fluorescence, the 488-nm excitation line was used; GFP fluorescence was collected with a 505- to 530-nm band-pass filter. All images in a single experiment were captured with the same setting. For root tissues, cells in the cortex or root cap layer of 4-d-old seedlings were consistently used for confocal microscopy observation. Imaging was performed using Zeiss LSM 880 (with Airyscan) or Leica SP8 confocal microscopes.

### Virus Inoculation

To generate the constructs with RFP-tag, TET3 fragments were cloned using the plasmid pDONR221-TET3, pDONR221-TET3ΔGCCKP, pDONR221-TET3^C174AC175A^, pDONR221-TET3^C221A^ as the templates. The resultant PCR products were further cloned into the destination vector by Gateway cloning (Supplementary Table 2).

For *cucumber mosaic virus* (CMV) infection assay, CMV was initially inoculated in *N. benthamiana* leaves. After 5–7 dpi, the infected tobacco leaves were collected and homogenized in PBS buffer (PH = 7.4). The homogenized mixtures were centrifuged and the virus enriched supernatants were transferred into fresh tubes. Equal volume of the virus (40 ng/μL) enriched supernatants was used to inoculate 3-week-old Arabidopsis leaves. Carborundum was firstly spread on these leave, 5 μL inoculum was rubbed on the leaf surface as previously described (Huang et al., 2019). CMV-infected TET leaves were collected for quantitative RT-PCR analysis of CMV Coat Protein (CP) gene abundance after 5 days of infection. The primers were listed in Supplementary Table 1 (Vinodhini et al., 2020).

For TRV infection assay, the *Agrobacterium* strain mixture harboring P19, *TRV1* and *TRV-GFP* (1:1:1) was diluted 15,000-fold and, respectively, co-infiltrated with *Agrobacterium* harboring 35S: RFP-TET3, 35S: RFP-TET3-ΔGCCKP, 35S: RFP-TET3^C174AC175A^, 35S: RFP-TET3^C221A^ or 35S: RFP (OD = 0.8) into tobacco leaves. At 5 day post-inoculation (dpi), TRV-GFP spread areas were observed and quantified, and the GFP fluorescence signal was imaged by Zeiss LSM880 confocal microscope.

### Quantitative Reverse Transcription-Polymerase Chain Reaction Analysis

Total RNA was extracted following the manufacturer’s instructions. cDNA for quantitative reverse transcription-PCR (q-PCR) analysis was synthesized using one-step genomic DNA removal and a cDNA synthesis kit (Transgen, Beijing, China). Q-PCR was performed using the MonAmpTM ChemoHS qPCR Mix (Monad, Wuhan, China). The primers used for qRT-PCR are listed in Supplementary Table 1. Quantification of the target gene was assessed by relative standard curves. The 2^–ΔΔCt^ algorithm was employed to quantify the relative gene expression (Livak and Schmittgen, 2001). The statistical significance of differences was calculated using GraphPad Prism 8 (Graphpad Software, Inc., San Diego, CA, United States) with Two-way ANOVA or 2-tailed Student’s *t*-test to obtain the *P*-value. Data were shown as mean ± SD of three biological replicates from one representative experiment. *P*-value of 0.05 or less was considered as significant differences.

### Statistical Analysis

Statistical data were analyzed in Graphpad Prism 8. Camera and confocal images were prepared with ImageJ.^6^ *P*-value of 0.05 or less was considered as significant differences.

### Cell Biological Quantification Method

Quantification of cytosol/PM signal ratio: the signal of TET and TET mutation variants was captured by Zeiss LSM880 with airyscan mode. Fluorescence of the cytosol, PM were individually measured by ImageJ. The fluorescence signal ratio of cytosol to PM was quantified as the signal ratio of (cytosol/PM) signal.

## Results

### *Arabidopsis thaliana* TETRASPANINs Are Plasma Membrane-Associated Proteins

**FIGURE 1:**
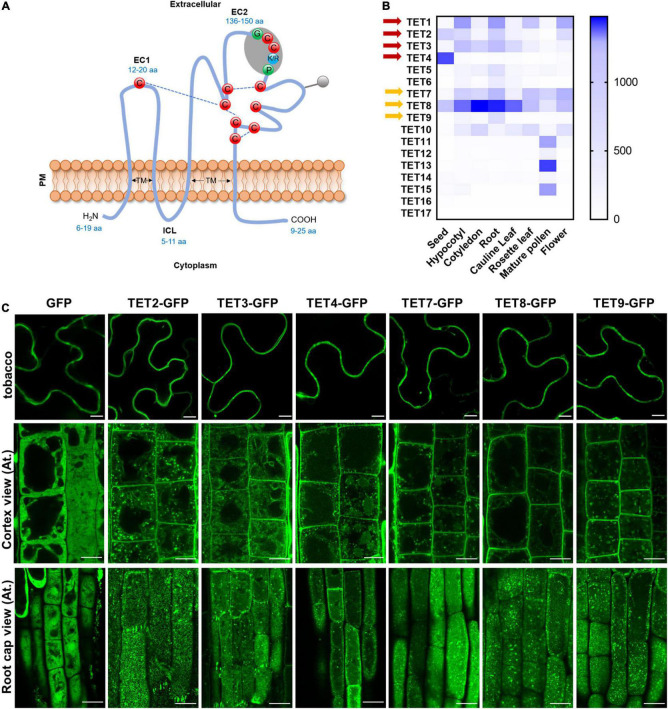
Tetraspanin (TET) expression patterns and subcellular localization. **(A)** Schematic representation plant tetraspanin topology. Red balls indicate conserved cysteine residues. Predicted disulfide bridges are shown with dashed blue lines. Gray shading represents the plant GCCK/RP motif in EC2. Numbers in blue indicate the range of amino acids. EC1 and EC2, small and large extracellular loop; ICL, intracellular loop; TM, transmembrane domains. **(B)** Heatmap representing expression profile of Arabidopsis tetraspanins in different organs. Gene expression data from Arabidopsis eFP Browser [Arabidopsis eFP Browser (utoronto.ca)]. Tetraspanin proteins discussed in this work are indicated by red and yellow arrows. **(C)** Subcellular localization of GFP-tagged tetraspanins in transient expression in *N. benthamiana* leaf epidermal cells (upper panels) and stable expression in *Arabidopsis thaliana* primary root cells (lower panels) (35S: GFP as control). The second and third rows show the GFP distribution of tetraspanins from cortex and root cap cells, respectively. Bars, 10 μm.

Arabidopsis contains 17 TET homologs with the typical transmembrane domains, extracellular loops, and conserved cysteine residues (Figure 1A and Supplementary Figure 1A). The tissue-specific expression assay revealed that TET genes were differentially expressed in various tissues, and TET1, 2, 3, 7, and 8 showed relatively higher expression in most tissues (Figure 1B). It suggested that TET1, 2, 3, 7, and 8 may play dominant roles in plant development. The un-rooted phylogenetic tree showed a close sequence similarity among TET1 and 2, TET3 and 4, as well as TET7, 8, 9 subclusters (Supplementary Figure 1B).

To understand the subcellular localization of Arabidopsis TETs, green fluorescent protein (GFP) was fused to the C-terminus of TET proteins (TET2, 3, 4, 7, 8, and 9), under the control of the constitutive Cauliflower mosaic virus 35S (CaMV-35S) promoter, which was based on the protein fusion criteria of previous reported TET proteins (Boavida et al., 2013). The empty vector (EV) 35S: GFP was used as a negative control. Transient expression of these constructs in tobacco epidermal leaves showed that these TETs distinctly accumulated at the periphery of tobacco epidermal cells (Figure 1C). To further confirm this result, we introduced the above TET-GFP fusion proteins in Arabidopsis plants and generated stable transgenic plants. Subcellular visualization showed that TET2, 3, 4, 7, 8, and 9 mainly targeted to the PM in Arabidopsis root and leaf cells (Figure 1C and Supplementary Figure 2B), and they were also found within endosomes or vacuole-like structures in the cytosol (Figure 1C). On the level of root cap cell layer, these TETs showed punctate distribution on root cell surface, which could be the foci of tetraspanin-enriched microdomains (Figure 1C).

To further examine the secretion and localization of TET proteins *in vivo*, we performed a plasmolysis assay on Arabidopsis root cells (Supplementary Figure 2A). The control GFP protein was found in both cytoplasm and nucleus (Supplementary Figure 2A). In contrast, AtTETs-GFP signals were clearly visualized in the membrane, co-shrinking with the plasmolyzed cells (Supplementary Figure 2A). The transient expression assay and stable transformation studies both revealed that AtTET proteins are preferentially associated with PM, which is in agreement with the conclusion in the previous study (Boavida et al., 2013).

### Tetraspanin-3 Association With Membrane Relies on Extracellular Loop and Transmembrane Regions

**FIGURE 2:**
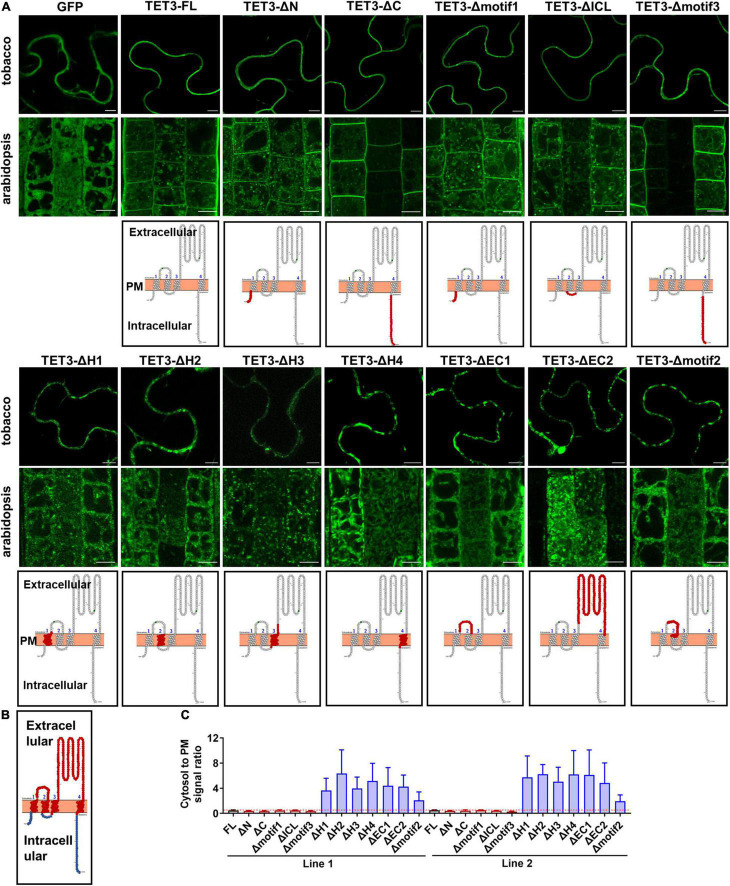
Extracellular and transmembrane regions are required for the plasma membrane (PM) targeting of AtTET3. **(A)** The first and second rows show the subcellular localization of specific regions removal fusion proteins of TET3 in *N. benthamiana*, *Arabidopsis thaliana*, respectively (35S:GFP as control). N and C, N- and C-terminal tail; EC1 and EC2, small and large extracellular loop; ICL, intracellular loop; H1–H4, transmembrane domains; motif1–3, motifs different from TET1, TET2, and TET7 to TET9. Bars, 10 μm. The third row shows the model of removal position of specific regions (red circle). **(B)** Red circles indicate the transmembranes and extracellular domains, blue circles indicate the intracellular domains of TET protein. **(C)** The fluorescence signal ratio of cytosol to PM was calculated by two independent lines of Arabidopsis transgenic plants (*n* = 144, 161, 86, 153, 192, 85, 139, 91, 130, 129, 140, 84, and 100). Error bar = S.D.

To identify regions which are possibly responsible for membrane association of TETs, we delineated the full-length TET3 coding sequence (as a representative) into 12 regions: N- and C-terminal regions and four TMs (H1-H4), ICL (Intracellular Loop), EC1 (small extracellular domain), and EC2 (small extracellular domain), motif1–3 (specific motifs in TET3), based on domain prediction (Figure 2B and Supplementary Figures 4A,B). The truncated TET3 variants with individual removal of the above 12 regions were fused with GFP. Further transient assay in tobacco leaves and stable transgenic assay in Arabidopsis root and leaf cells both showed that the removal of N- and C-terminal, ICL, motif1, and motif3 was not able to change the membrane targeting of TET3 (Figure 2A and Supplementary Figure 3). In contrast, deletion of H1-H4, EC1, EC2 or motif2 were all deprived of the membrane association ability of TET3 (Figure 2A and Supplementary Figure 3). Quantification of cytosol versus PM signal ratio in two independent transgenic Arabidopsis lines confirmed that the removal of H1–H4, EC1, EC2 or motif2 results in strong residence of TET3 within the cytosol (Figure 2C).

Altogether, our data demonstrated that the transmembrane domains and extracellular loops are necessary to drive TET proteins to the PM, whereas the intracellular domains restrict TET distribution within the cytosol.

### GCCKP Motif Is Involved in Efficient Targeting of Tetraspanin to the Plasma Membrane

**FIGURE 3:**
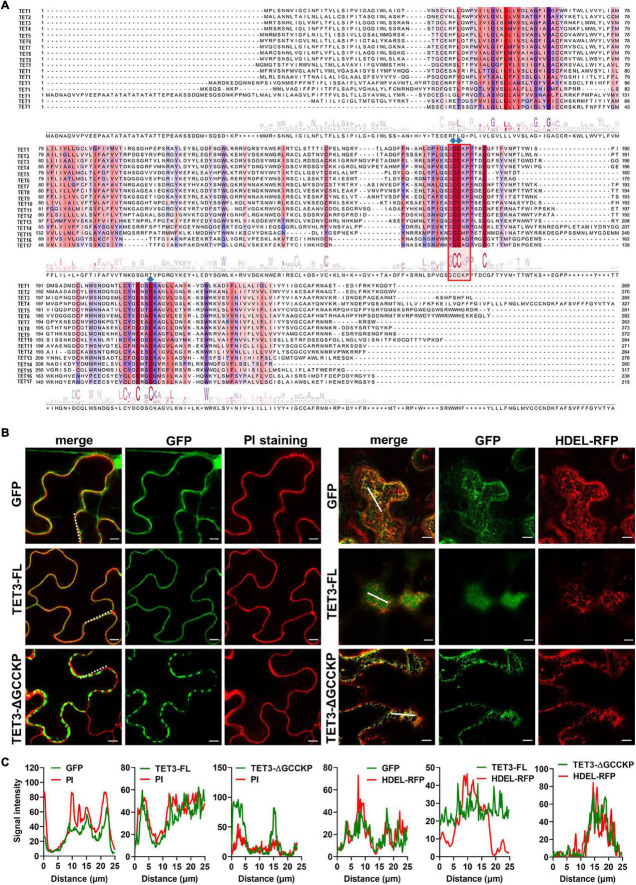
Contribution of GCCKP motif for TET3 subcellular location. **(A)** Multiple alignment of the AtTET family was performed by Clustal X. Conserved residues are highlighted in colors. Red box indicates the conserved GCCK/RP motif among Arabidopsis tetraspanins. Conserved Cys residues discussed in this work are ndicated by blue arrows. **(B)** The TET3 or TET3-ΔGCCKP proteins fused to GFP were transiently expressed in *N. benthamiana* leaves (35S: GFP as control). Left panel shows colocalization of TET3-GFP or TET3ΔGCCKP-GFP proteins with PI-stained PM. Right panels show the colocalization of TET3-GFP or TET3ΔGCCKP-GFP proteins with HDEL-RFP. The merged fluorescence of marker and of GFP is shown at the left pictures. Bars, 10 μm. **(C)** Colocalization fluorescence signal profile chart was generated based on the white dot and solid line from Figure 2B.

The sequence alignment of 17 AtTETs revealed that the “GCCK/RP” motif in EC2 was conserved in most TETs but few variants in TET14, 15, 16, and 17 proteins (Figure 3A). Moreover, a series of conserved cysteine residues were present in EC2 of TETs, which are predicted to serve as adaptors for partner interactions (Charrin et al., 2002; Yang et al., 2004; Figure 3A).

To further understand the contribution of the “GCCK/RP” motif in TET distribution, this motif was removed and the truncated TET3 variant was further fused with GFP tag. Compared with the intact full-length TET3 that co-localized with PI-stained PM, TET3ΔGCCKP displayed punctate structure and PM disassociation, revealing that TET3ΔGCCKP was abolished from the membrane (Figures 3B,C). In addition, TET3ΔGCCKP showed a co-localization with the defined endoplasmic reticulum (ER) marker, HDEL-RFP (Figures 3B,C). To rule out the possibility that the “GCCK/RP” motif is an exception for TET3, we also tested the contribution of the “GCCK/RP” motif for other TET proteins, TET4 and TET7. Removal of “GCCK/RP” in both TET4 and TET7 showed a consistent distribution of TET4ΔGCCKP and TET7ΔGCCKP to ER (Supplementary Figures 5A,B). Therefore, the conserved “GCCK/RP” motif in EC2 anchors TET proteins to the PM.

### Cysteine Residues Are Sufficient to Control Membrane Association of Tetraspanin

As already mentioned, the “GCCK/RP” motif that presents in EC2 is highly conserved among plant TETs, meanwhile, EC2 contains up to nine strictly conserved cysteine residues which could serve as adaptors to influence protein-TET interactions (Boavida et al., 2013; Cunha et al., 2017). Previous studies have reported that some intracellular juxtamembrane cysteine residues undergo palmitoylation, possibly required for the association between TETs and their interactors (Berditchevski et al., 2002; Charrin et al., 2002; Yang et al., 2002; Delandre et al., 2009). Palmitoylation is a post-translational protein modification type, affecting protein stability, and sorting, etc. (Greaves and Chamberlain, 2007; Linder and Deschenes, 2007). In animals, TET palmitoylation modulates the organization of the integrin-TEM complex during cell adhesion (Berditchevski et al., 2002). To understand the possible function of cysteine residues in plant TETs, we first predict the palmitoylation sites of TET3. Palmitoylation assay showed that the palmitoylation sites were automatically clustered into three clusters by different thresholds (high, medium, low) of peptides similarity (Zhou et al., 2006). The cysteine residues (in 174, 175, and 221 sites) of TET3 potentially harbor relatively high palmitoylation ability (Supplementary Figure 6A). Apparently, it is noteworthy to understand the role of these cysteine residues in TET function.

**FIGURE 4:**
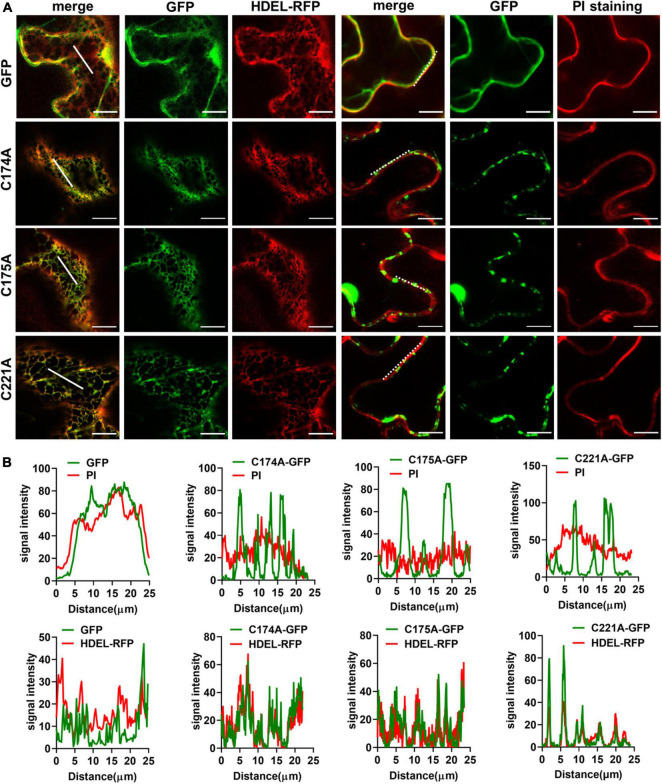
Conserved Cysteine residues are required for Tetraspanin (TET) targeting to PM. **(A)** The conserved cysteine residues (individual at the positions of 174, 175, and 221) of TET3 protein were, respectively, substituted by alanine (35S: GFP as control), and the artificial point mutated TET3 proteins fused with GFP were transiently expressed in *N. benthamiana* leaves. Overlapping fluorescence spectra analysis of GFP and HDEL-RFP signal or PI-stained PM is shown. Bars, 10 μm. **(B)** Colocalization fluorescence signal profile chart was generated based on the white dot and solid line from panel **(A)**.

We then carried out site-directed mutagenesis based on the above prediction (Figure 3A and Supplementary Figure 6A), switching cysteine to be alanine. The resultant TET3-GFP variants were introduced in tobacco leaves. Co-localization of TET3^C174A^-GFP, TET3^C175A^-GFP, TET3^C221A^-GFP with HDEL-RFP marker or PI dye showed that these TET3 variants were all distributed at mesh-like ER structures, instead of PM (Figures 4A,B). Replacement of the single cysteine residue already changes the distribution of TET3, indicating the central role of cysteine residues in control of TET distribution. Strikingly, either the cysteine residues located within (174, 175 sites) or out (221 site) of GCCK/RP motif in EC2 were all able to influence membrane association ability of TET, suggesting that these conserved cysteine residues are important for a functional TET3 protein, independent from GCCK/RP motif.

Interestingly, TETs distributed in a foci pattern on the cell surface, suggesting a possible correlation with plasmodesmata (PD), which direct cell-to-cell communication between neighboring cells (Fernandez-Calvino et al., 2011; Wang et al., 2015). We thus co-expressed TET3 with a known PD maker, PDLP5-RFP in tobacco leaves. Co-localization analysis confirmed that TET3 targets to PD (Supplementary Figure 7). Compared with the high association of TET3 with PD, the TET mutation variants without GCCK/RP or cysteine residues showed no distinct PD subcellular localizations (Supplementary Figure 7).

Altogether, these results proved the importance of the conversed cysteine residues in governing the distribution of TET proteins to the PM.

### Tetraspanin-Mediated Virus Movement Requires GCCK/RP Motif and Cysteine Residues

**FIGURE 5:**
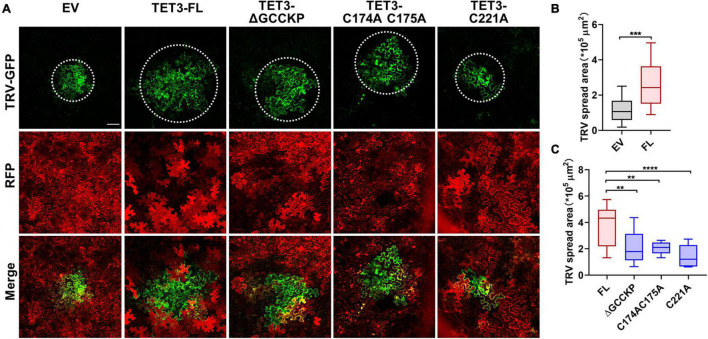
Tetraspanin-3 (TET3) facilitates cell-to-cell movement of TRV in *N. benthamiana*. **(A)** Cell-to-cell movement of the TRV-GFP in empty vector (EV, 35S: RFP as control), full length (FL), GCCK/RP motif knockout, and cysteine residues mutated TET transiently expressing *N. benthamiana* leaves. Bars, 100 μm. **(B,C)** Measurement of TRV-GFP cell-to-cell movement area. TRV-GFP co-infiltration with empty vector (35S: RFP), the GFP spread area was observed and quantified at 5 dpi (*n* = 24, 21, 23, 25, 25, and 23). Error bar = S.D. *P*-values were determined by two-tailed Student’s *t*-test assuming equal variances **(B)** and one-way analysis of variance (ANOVA) with the Turkey *post-hoc* test **(C)** (**p < 0.01; ***p < 0.001; ****p < 0.0001).

Accumulating evidence indicates that TET is essential for pathogen invasion *via* a possible interaction between TET and specific viral receptors within TET-enriched microdomains (van Spriel and Figdor, 2010). Hence, TET can promote virus binding, coordinate virus trafficking and fusion events (van Spriel and Figdor, 2010). To understand the genetic correlation and biological significance of TET distribution during virus infection, we examined the ability of intact TET3 or TET3 variants on the cell-to-cell movement of *tobacco rattle virus* (TRV) in tobacco leaves. Compared with the control (empty vector, EV), intact TET3 protein (TET3-FL) significantly promoted virus spread by approximately twofold (Figure 5). The variants without GCCK/RP motif (TET3-ΔGCCKP-RFP) or cysteine residues variants (TET3^C174AC175A^-RFP, TET3^C221A^-RFP) which abolished membrane-association of TET3 all showed significant attenuation of TRV spread within tobacco leave cells (Figure 5 and Supplementary Figure 8A). These results revealed that virus movement requires the assistance of membrane-associated TETs, and GCCK/RP motif and cysteine residues are all involved in TET functionality during virus infection.

**FIGURE 6:**
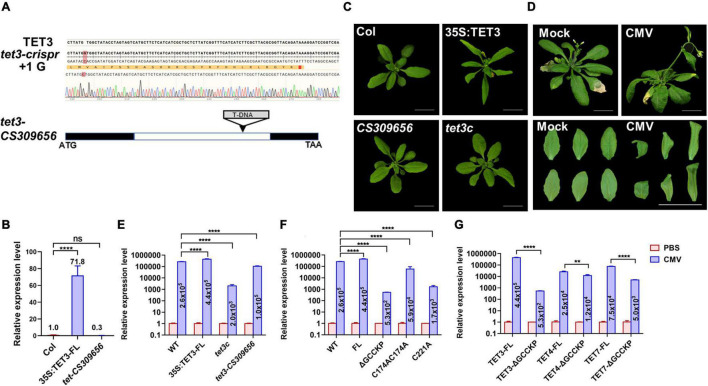
Tetraspanin-3 (TET3) protein promotes CMV infection. **(A)** CRISPR-cas9-based TET3 mutantgenesis (*tet3c*) and T-DNA-directed TET3 knockdown mutant (*CS309656*) are shown. Black boxes represent the exons and white box represents the introns. **(B)** Transcript level of TET3 gene in overexpression and mutant lines, *P*-values were determined one-way analysis of variance (ANOVA) with the Turkey *post-hoc* test (****P < 0.0001; ns, not significant). The average transcript level of TET3 was quantified from three times of qRT-PCR (*n* = 3). **(C)** Leaf phenotype of 3-week-old 35S:TET3 and tet3 mutants were shown. **(D)** CMV infection symptoms in Arabidopsis WT plants at 5 dpi were shown. Scale bars = 2 cm. **(E–G)** Q-PCR analysis of transcript levels for AtTET in overexpression of intact or mutation variants of TET proteins were shown at 5 dpi. The numbers in the histogram represent the relative transcription value of the target genes (AtTET3, AtTET4, and AtTET7) after CMV infection. Error bars represent SD of three independent replicates. *P*-values were determined by two-way analysis of variance (ANOVA) **(E,F)** or two-tailed Student’s *t*-test assuming equal **(G)** (**p < 0.01; ****p < 0.0001).

To verify the functionality of TET3 in virus infection, we generated Arabidopsis *tet3* mutant by designing a CRISPRCas9–mediated TET3 gene deficiency, termed *tet3c* (Figure 6A). Meanwhile, we obtained a T-DNA insertion knockdown mutant of *tet3-CS309656*, with the insertion site in TET3 intron (Figures 6A,B). Further phenotypic analysis revealed that *35s:TET3* plants exhibited much slender and curled leaves than WT; however, *tet3* mutants were comparable as WT, which may be caused by the functional redundancy (Figure 6C).

To prove the hypothesis that TET3 promotes viral infection, we inoculated Arabidopsis leaves with *cucumber mosaic virus* (CMV). At 5 days post-inoculation (dpi), WT plants exhibited systemic virus infection symptoms including twisted stems and curly leaves (Figure 6D). We then applied qRT-PCR to test the transcriptional level of a CMV coat protein (CP) which reflected the infection efficiency of CMV in *tet3* mutants, overexpression of TET3 intact protein and TET3 variants. Two independent *tet3* mutants both significantly decreased the accumulation of CMV compared with WT; *35S:TET3* promoted CMV infection to twofold, in comparison of WT. Moreover, CMV infection efficiency in TET3 ΔGCCKP and cysteine residues variants was significantly disrupted, in comparison of intact TET3 form (Figures 6E–G). Moreover, TET4 and TET7 variants without GCCKP motif also disrupted CMV infection efficiency, in agreement with the property of TET3 protein (Figures 6E–G). The above CMV infection data in Arabidopsis plants supported the essential role of TET3 protein for virus infection.

Together, these data consistently showed that membrane-associated TET is required for virus infection and that both GCCK/RP motifs and cysteine residues are involved in TET function during viral infection.

## Discussion

As a highly conserved transmembrane protein, TET is ubiquitous in eukaryotic organisms. In animals and fungi, increasing evidence indicates that TETs act as master organizers of the PM by forming TEMs through the interactions of TETs with other membrane proteins, which were “hijacked” by viruses as a gateway for entry and egress (Florin and Lang, 2018). Our study further clarifies the importance of plant TETs for virus spread, which is dependent on the specific GCCK/RP motif and the conserved cysteine residues.

Animal and plant TETs share a common structure consisting of four transmembrane domains, a small extracellular loop, and a large extracellular loop. The major extracellular loop EC2 accounts for the length of TETs, suggesting the EC2 region is central for TETs function. Indeed, EC2 of animal TETs could be recognized by most TET-specific monoclonal antibodies (Levy and Shoham, 2005). Compelling evidence indicates that the large extracellular loop (LEL) of mast cell-expressed TET proteins [Cluster of differentiation (CDs), such as CD9, CD63, CD81, CD82, CD151] plays a key role in the route of pathogens infection (Pileri et al., 1998; Ho et al., 2006; Mazurov et al., 2007; Shanmukhappa et al., 2007; Singethan and Schneider-Schaulies, 2008). Especially, blocking the activity of large extracellular loop domain of *Fenneropenaeus chinensis* tetraspanin-3 by anti-LEL antibody significantly inhibit the infection of white spot syndrome virus in Chinese shrimp (Gui et al., 2012). In animals, the EC2 domain of TETs may be relevant for the protein complex formation with other proteins (Hemler, 2001, 2003; Stipp et al., 2003). In plants, single amino acid changes in the EC2 or deletion of C-terminal tail of *TET1/TRN2* gene caused severe developmental defects, like a short primary root, small leaves, and a dwarf architecture (Cnops et al., 2006). Given the great importance of EC2 in TET function, EC2 attracted much attention as a promising tool to investigate the specific role of different TETs (Grove et al., 2017). Moreover, TET antibodies that preferentially target EC2 provide a therapeutic means to inhibit virus movement (Stipp et al., 2003).

Instead of a highly conserved CCG motif within the EC2 domain in animals and metazoans TETs, plant TETs contain a signature “GCCK/RP” motif (Stipp et al., 2003; Wang et al., 2012). “GCCK/RP” motif can be traced back to the ancient moss *Physcomitrium patens* and vascular plant *Selaginella moellendorffii*, suggesting that this motif emerged early in plant ancestors (DeSalle et al., 2010). In animals, two cysteine residues in the CCG motif form two intramolecular disulfide bridges with the other two cysteine residues (Kitadokoro et al., 2001). Nevertheless, plant TETs have nine, rather than four, six, or eight conserved cysteine residues (Reimann et al., 2017), making the protein structure even complicated than animal TETs. In our study, mutation of cysteine residues 174, 175, 221 or removal of GCCKP motif abolished membrane association of TET proteins, leading to its accumulation within ER. This suggests that these residues may participate in in protein folding. Indeed, the more cysteine residues in a protein, the higher possibility to form disulfide bonds (Fu et al., 2020). Any two cysteine residues within a protein are able to form a disulfide bond (Fu et al., 2020). Disulfide bonds between conserved cysteines produces a sub-loop structure (Boavida et al., 2013; van Deventer et al., 2017). In addition, disulfide bonds between the large and small loops, which are essential for TET-TET interactions, could stabilize their interaction in a redox-dependent fashion or affect the binding affinity of cholesterol or gangliosides with membrane. Misfolding of protein often leads to the loss of its biological function (Thomas et al., 1995; Kim et al., 1996; Harper and Lansbury, 1997; Cabral et al., 2001). In plants, missense mutations in these cysteine residues in *trn2-2* and *trn2-3* alleles may affect protein folding due to a defect in disulfide bridges and thereby inhibit TET function (Cnops et al., 2006).

Besides, post-translation modifications, such as glycosylphosphatidylinositol (GPI)-anchoring, palmitoylation may also contribute to protein PM localization (Galian et al., 2012; Gui et al., 2015; Kumar et al., 2016). Three putative palmitoylation sites, Cys174, Cys175, Cys221 in AtTET3 were predicted with higher scores of palmitoylation. All of these variants display an ER localization. One plausible explanation would be that these cysteine residues provide sites for palmitoylation that all contribute to the membrane association of TET to the PM. Palmitoylation, also called S-acylation, mediates protein association with membranes, which has been verified in remorin proteins and other membrane-associated proteins (Konrad et al., 2014; Hurst and Hemsley, 2015). In recent years, palmitoylation in plants has begun to come of age. Some novel palmitoylated proteins have been identified, including the membrane microdomain organizing proteins and Raf-like MAP kinases (Hemsley et al., 2013). Palmitoylation was supposed to play a critical role in the microdomain localization of membrane-resident proteins (Blaskovic et al., 2013). A previous report showed that a tobacco remorin protein, NbREM1 is modified by S-acylation of cysteine at the C-terminal, and the remorin variant with a single cysteine mutation loses the ability to associate with PM (Fu et al., 2018). Additionally, single cysteine residue at the C terminus is also required for the PM localization of *Medicago truncatula* and *A. thaliana* remorins (Konrad et al., 2014). Similarly, the palmitoylation of the juxtamembrane cysteine residues is present in many TETs, including CD9, CD63, CD81, and CD151 (Charrin et al., 2002). Mutagenesis of cysteine residue causes palmitoylation deficiency and altered cellular distribution of CD151 (Yang et al., 2002). Importantly, protein palmitoylation contributes not only to membrane association but also to the regulation of protein-protein interactions and TET web formation (Stipp et al., 2003; Blaskovic et al., 2013). Loss of palmitoylation resulted in decreased lateral associations of CD151 and CD9 with other TETs (Berditchevski et al., 2002; Charrin et al., 2002; Yang et al., 2002). Our study also proved that a single cysteine residue in or neighboring to the “GCCK/RP” motif is sufficient to change TET3 targeting to the PM (Figure 4). Despite all these evidences revealing the importance of palmitoylation at cysteine residues for assembly and maintenance of membrane nanodomains, sole palmitoylation modification might be not sufficient to establish the interactive network of TETs with other cell-surface proteins (Sincock et al., 1999; Claas et al., 2001; Konrad et al., 2014). Thus, further clarification of such residues would be of high relevance for elucidation of the regulatory mechanisms of TET proteins, but also be a general mechanism of other membrane-associated proteins.

## Data Availability Statement

The datasets presented in this study can be found in online repositories. The names of the repository/repositories and accession number(s) can be found in the article/Supplementary Material.

## Author Contributions

TZ and YS designed and performed the experiments. TZ and XC assisted with the experimental procedures and the data analysis. TZ wrote the manuscript. XC edited the manuscript and provided the supervision, funding, and reagents. All authors contributed to the article and approved the submitted version.

## Conflict of Interest

The authors declare that the research was conducted in the absence of any commercial or financial relationships that could be construed as a potential conflict of interest.

## Publisher’s Note

All claims expressed in this article are solely those of the authors and do not necessarily represent those of their affiliated organizations, or those of the publisher, the editors and the reviewers. Any product that may be evaluated in this article, or claim that may be made by its manufacturer, is not guaranteed or endorsed by the publisher.

## Footnotes

1
www.invitrogen.com

2
http://www.arabidopsis.org

3
https://www.uniprot.org/

4
http://wlab.ethz.ch/protter/start/

5
https://meme-suite.org/meme/

6
http://imagej.nih.gov/ij/

## References

[B1] Berditchevski F. Odintsova E. (1999). Characterization of integrin-tetraspanin adhesion complexes: role of tetraspanins in integrin signaling. *J. Cell Biol.* 146 477–492. 10.1083/jcb.146.2.477 10427099 PMC2156181

[B2] Berditchevski F. Odintsova E. Sawada S. Gilbert E. (2002). Expression of the palmitoylation-deficient CD151 weakens the association of alpha 3 beta 1 integrin with the tetraspanin-enriched microdomains and affects integrin-dependent signaling. *J. Biol. Chem.* 277 36991–37000. 10.1074/jbc.M205265200 12110679

[B3] Blaskovic S. Blanc M. van der Goot F. G. (2013). What does S-palmitoylation do to membrane proteins? *FEBS J.* 280 2766–2774. 10.1111/febs.12263 23551889

[B4] Boavida L. C. Qin P. Broz M. Becker J. D. McCormick S. (2013). *Arabidopsis* tetraspanins are confined to discrete expression domains and cell types in reproductive tissues and form homo- and heterodimers when expressed in yeast. *Plant Physiol.* 163 696–712. 10.1104/pp.113.216598 23946353 PMC3793051

[B5] Burckhardt C. J. Greber U. F. (2009). Virus movements on the plasma membrane support infection and transmission between cells. *PLoS Pathog.* 5:e1000621. 10.1371/journal.ppat.1000621 19956678 PMC2777510

[B6] Cabral C. M. Liu Y. Sifers R. N. (2001). Dissecting glycoprotein quality control in the secretory pathway. *Trends Biochem. Sci.* 26 619–624. 10.1016/s0968-0004(01)01942-9 11590015

[B7] Cai Q. Qiao L. Wang M. He B. Lin F. M. Palmquist J. (2018). Plants send small RNAs in extracellular vesicles to fungal pathogen to silence virulence genes. *Science* 360 1126–1129. 10.1126/science.aar4142 29773668 PMC6442475

[B8] Charrin S. Manié S. Oualid M. Billard M. Boucheix C. Rubinstein E. (2002). Differential stability of tetraspanin/tetraspanin interactions: role of palmitoylation. *FEBS Lett.* 516 139–144. 10.1016/s0014-5793(02)02522-x 11959120

[B9] Chiu W. H. Chandler J. Cnops G. Van Lijsebettens M. Werr W. (2007). Mutations in the TORNADO2 gene affect cellular decisions in the peripheral zone of the shoot apical meristem of *Arabidopsis thaliana*. *Plant Mol. Biol.* 63 731–744. 10.1007/s11103-006-9105-z 17351828

[B10] Claas C. Stipp C. S. Hemler M. E. (2001). Evaluation of prototype transmembrane 4 superfamily protein complexes and their relation to lipid rafts. *J. Biol. Chem.* 276 7974–7984. 10.1074/jbc.M008650200 11113129

[B11] Cnops G. Neyt P. Raes J. Petrarulo M. Nelissen H. Malenica N. (2006). The TORNADO1 and TORNADO2 genes function in several patterning processes during early leaf development in Arabidopsis thaliana. *Plant Cell* 18 852–866. 10.1105/tpc.105.040568 16531491 PMC1425859

[B12] Cnops G. Wang X. Linstead P. Van Montagu M. Van Lijsebettens M. Dolan L. (2000). Tornado1 and tornado2 are required for the specification of radial and circumferential pattern in the *Arabidopsis* root. *Development* 127 3385–3394. 10887093 10.1242/dev.127.15.3385

[B13] Cunha E. S. Sfriso P. Rojas A. L. Roversi P. Hospital A. Orozco M. (2017). Mechanism of structural tuning of the hepatitis C virus human cellular receptor CD81 large extracellular loop. *Structure* 25 53–65. 10.1016/j.str.2016.11.003 27916518

[B14] Delandre C. Penabaz T. R. Passarelli A. L. Chapes S. K. Clem R. J. (2009). Mutation of juxtamembrane cysteines in the tetraspanin CD81 affects palmitoylation and alters interaction with other proteins at the cell surface. *Exp. Cell Res.* 315 1953–1963. 10.1016/j.yexcr.2009.03.013 19328198 PMC2785499

[B15] DeSalle R. Mares R. Garcia-España A. (2010). Evolution of cysteine patterns in the large extracellular loop of tetraspanins from animals, fungi, plants and single-celled eukaryotes. *Mol. Phylogenet. Evol.* 56 486–491. 10.1016/j.ympev.2010.02.015 20171294

[B16] Fernandez-Calvino L. Faulkner C. Walshaw J. Saalbach G. Bayer E. Benitez-Alfonso Y. (2011). *Arabidopsis* plasmodesmal proteome. *PLoS One* 6:e18880. 10.1371/journal.pone.0018880 21533090 PMC3080382

[B17] Ferrari S. Galletti R. Denoux C. De Lorenzo G. Ausubel F. M. Dewdney J. (2007). Resistance to *Botrytis cinerea* induced in *Arabidopsis* by elicitors is independent of salicylic acid, ethylene, or jasmonate signaling but requires PHYTOALEXIN DEFICIENT3. *Plant Physiol.* 144 367–379. 10.1104/pp.107.095596 17384165 PMC1913806

[B18] Florin L. Lang T. (2018). Tetraspanin assemblies in virus infection. *Front. Immunol.* 9:1140. 10.3389/fimmu.2018.01140 29887866 PMC5981178

[B19] Fu J. Gao J. Liang Z. Yang D. (2020). PDI-regulated disulfide bond formation in protein folding and biomolecular assembly. *Molecules* 26:171. 10.3390/molecules26010171 33396541 PMC7794689

[B20] Fu S. Xu Y. Li C. Li Y. Wu J. Zhou X. (2018). Rice stripe virus interferes with s-acylation of remorin and induces its autophagic degradation to facilitate virus infection. *Mol. Plant* 11 269–287. 10.1016/j.molp.2017.11.011 29229567

[B21] Galian C. Björkholm P. Bulleid N. von Heijne G. (2012). Efficient glycosylphosphatidylinositol (GPI) modification of membrane proteins requires a C-terminal anchoring signal of marginal hydrophobicity. *J. Biol. Chem.* 287 16399–16409. 10.1074/jbc.M112.350009 22431723 PMC3351287

[B22] Garcia-España A. DeSalle R. (2009). Intron sliding in tetraspanins. *Commun. Integr. Biol.* 2 394–395. 10.4161/cib.2.5.8760 19907697 PMC2775230

[B23] Greaves J. Chamberlain L. H. (2007). Palmitoylation-dependent protein sorting. *J. Cell Biol.* 176 249–254. 10.1083/jcb.200610151 17242068 PMC2063950

[B24] Grove J. Hu K. Farquhar M. J. Goodall M. Walker L. Jamshad M. (2017). A new panel of epitope mapped monoclonal antibodies recognising the prototypical tetraspanin CD81. *Wellcome Open Res.* 2:82. 10.12688/wellcomeopenres.12058.1 29090272 PMC5657224

[B25] Gui J. Zheng S. Shen J. Li L. (2015). Grain setting defect1 (GSD1) function in rice depends on S-acylation and interacts with actin 1 (OsACT1) at its C-terminal. *Front. Plant Sci.* 6:804. 10.3389/fpls.2015.00804 26483819 PMC4590517

[B26] Gui L. Wang B. Li F. H. Sun Y. M. Luo Z. Xiang J. H. (2012). Blocking the large extracellular loop (LEL) domain of FcTetraspanin-3 could inhibit the infection of white spot syndrome virus (WSSV) in Chinese shrimp, *Fenneropenaeus chinensis*. *Fish Shellfish Immunol.* 32 1008–1015. 10.1016/j.fsi.2012.02.022 22406449

[B27] Harper J. D. Lansbury P. T. Jr. (1997). Models of amyloid seeding in Alzheimer’s disease and scrapie: mechanistic truths and physiological consequences of the time-dependent solubility of amyloid proteins. *Annu. Rev. Biochem.* 66 385–407. 10.1146/annurev.biochem.66.1.385 9242912

[B28] Hassuna N. Monk P. N. Moseley G. W. Partridge L. J. (2009). Strategies for targeting tetraspanin proteins: potential therapeutic applications in microbial infections. *BioDrugs* 23 341–359. 10.2165/11315650-000000000-00000 19894777 PMC7100176

[B29] Heinlein M. (2015). Plasmodesmata: channels for viruses on the move. *Methods Mol. Biol.* 1217 25–52. 10.1007/978-1-4939-1523-1_2 25287194

[B30] Hemler M. E. (2001). Specific tetraspanin functions. *J. Cell Biol.* 155 1103–1107. 10.1083/jcb.200108061 11756464 PMC2199333

[B31] Hemler M. E. (2003). Tetraspanin proteins mediate cellular penetration, invasion, and fusion events and define a novel type of membrane microdomain. *Annu. Rev. Cell Dev. Biol.* 19 397–422. 10.1146/annurev.cellbio.19.111301.153609 14570575

[B32] Hemler M. E. (2005). Tetraspanin functions and associated microdomains. *Nat. Rev. Mol. Cell Biol.* 6 801–811. 10.1038/nrm1736 16314869

[B33] Hemsley P. A. Weimar T. Lilley K. Dupree P. Grierson C. (2013). Palmitoylation in plants: new insights through proteomics. *Plant Signal. Behav.* 8:e25209. 10.4161/psb.25209 23759553 PMC3999067

[B34] Ho S. H. Martin F. Higginbottom A. Partridge L. J. Parthasarathy V. Moseley G. W. (2006). Recombinant extracellular domains of tetraspanin proteins are potent inhibitors of the infection of macrophages by human immunodeficiency virus type 1. *J. Virol.* 80 6487–6496. 10.1128/jvi.02539-05 16775336 PMC1488983

[B35] Huang D. Sun Y. Ma Z. Ke M. Cui Y. Chen Z. (2019). Salicylic acid-mediated plasmodesmal closure via Remorin-dependent lipid organization. *Proc. Natl. Acad. Sci. U.S.A.* 116 21274–21284. 10.1073/pnas.1911892116 31575745 PMC6800329

[B36] Hurst C. H. Hemsley P. A. (2015). Current perspective on protein S-acylation in plants: more than just a fatty anchor? *J. Exp. Bot.* 66 1599–1606. 10.1093/jxb/erv053 25725093

[B37] Jacobson K. Liu P. Lagerholm B. C. (2019). The lateral organization and mobility of plasma membrane components. *Cell* 177 806–819. 10.1016/j.cell.2019.04.018 31051105 PMC6541401

[B38] Jimenez-Jimenez S. Hashimoto K. Santana O. Aguirre J. Kuchitsu K. Cardenas L. (2019). Emerging roles of tetraspanins in plant inter-cellular and inter-kingdom communication. *Plant Signal. Behav.* 14:e1581559. 10.1080/15592324.2019.1581559 30829110 PMC6512927

[B39] Kim P. S. Kwon O. Y. Arvan P. (1996). An endoplasmic reticulum storage disease causing congenital goiter with hypothyroidism. *J. Cell Biol.* 133 517–527. 10.1083/jcb.133.3.517 8636228 PMC2120816

[B40] Kitadokoro K. Bordo D. Galli G. Petracca R. Falugi F. Abrignani S. (2001). CD81 extracellular domain 3D structure: insight into the tetraspanin superfamily structural motifs. *EMBO J.* 20 12–18. 10.1093/emboj/20.1.12 11226150 PMC140195

[B41] Konrad S. S. Popp C. Stratil T. F. Jarsch I. K. Thallmair V. Folgmann J. (2014). S-acylation anchors remorin proteins to the plasma membrane but does not primarily determine their localization in membrane microdomains. *New Phytol.* 203 758–769. 10.1111/nph.12867 24897938

[B42] Kovalenko O. V. Metcalf D. G. DeGrado W. F. Hemler M. E. (2005). Structural organization and interactions of transmembrane domains in tetraspanin proteins. *BMC Struct. Biol.* 5:11. 10.1186/1472-6807-5-11 15985154 PMC1190194

[B43] Kumar M. Wightman R. Atanassov I. Gupta A. Hurst C. H. Hemsley P. A. (2016). S-Acylation of the cellulose synthase complex is essential for its plasma membrane localization. *Science* 353 166–169. 10.1126/science.aaf4009 27387950

[B44] Kumar S. Stecher G. Li M. Knyaz C. Tamura K. (2018). MEGA X: molecular evolutionary genetics analysis across computing platforms. *Mol. Biol. Evol.* 35 1547–1549. 10.1093/molbev/msy096 29722887 PMC5967553

[B45] Lambou K. Tharreau D. Kohler A. Sirven C. Marguerettaz M. Barbisan C. (2008). Fungi have three tetraspanin families with distinct functions. *BMC Genomics* 9:63. 10.1186/1471-2164-9-63 18241352 PMC2278132

[B46] Levy S. Shoham T. (2005). The tetraspanin web modulates immune-signalling complexes. *Nat. Rev. Immunol.* 5 136–148. 10.1038/nri1548 15688041

[B47] Lieber D. Lora J. Schrempp S. Lenhard M. Laux T. (2011). *Arabidopsis* WIH1 and WIH2 genes act in the transition from somatic to reproductive cell fate. *Curr. Biol.* 21 1009–1017. 10.1016/j.cub.2011.05.015 21658947

[B48] Linder M. E. Deschenes R. J. (2007). Palmitoylation: policing protein stability and traffic. *Nat. Rev. Mol. Cell Biol.* 8 74–84. 10.1038/nrm2084 17183362

[B49] Livak K. J. Schmittgen T. D. (2001). Analysis of relative gene expression data using real-time quantitative PCR and the 2(-Delta Delta C(T)) Method. *Methods* 25 402–408. 10.1006/meth.2001.1262 11846609

[B50] Maecker H. T. Todd S. C. Levy S. (1997). The tetraspanin superfamily: molecular facilitators. *FASEB J.* 11 428–442. 9194523

[B51] Marsh M. Helenius A. (2006). Virus entry: open sesame. *Cell* 124 729–740. 10.1016/j.cell.2006.02.007 16497584 PMC7112260

[B52] Martin F. Roth D. M. Jans D. A. Pouton C. W. Partridge L. J. Monk P. N. (2005). Tetraspanins in viral infections: a fundamental role in viral biology? *J. Virol.* 79 10839–10851. 10.1128/jvi.79.17.10839-10851.2005 16103137 PMC1193642

[B53] Mazurov D. Heidecker G. Derse D. (2007). The inner loop of tetraspanins CD82 and CD81 mediates interactions with human T cell lymphotrophic virus type 1 Gag protein. *J. Biol. Chem.* 282 3896–3903. 10.1074/jbc.M607322200 17166843

[B54] Miyauchi K. Kim Y. Latinovic O. Morozov V. Melikyan G. B. (2009). HIV enters cells via endocytosis and dynamin-dependent fusion with endosomes. *Cell* 137 433–444. 10.1016/j.cell.2009.02.046 19410541 PMC2696170

[B55] Mudhakir D. Harashima H. (2009). Learning from the viral journey: how to enter cells and how to overcome intracellular barriers to reach the nucleus. *AAPS J.* 11 65–77. 10.1208/s12248-009-9080-9 19194803 PMC2664881

[B56] Nathalie L.-C. Bouhidel K. (2014). Plasma membrane protein trafficking in plant-microbe interactions: a plant cell point of view. *Front. Plant Sci.* 5:735. 10.3389/fpls.2014.00735 25566303 PMC4273610

[B57] Pileri P. Uematsu Y. Campagnoli S. Galli G. Falugi F. Petracca R. (1998). Binding of hepatitis C virus to CD81. *Science* 282 938–941. 10.1126/science.282.5390.938 9794763

[B58] Reimann R. Kost B. Dettmer J. (2017). TETRASPANINs in plants. *Front. Plant Sci.* 8:545. 10.3389/fpls.2017.00545 28458676 PMC5394113

[B59] Ren J. Wen L. Gao X. Jin C. Xue Y. Yao X. (2008). CSS-Palm 2.0: an updated software for palmitoylation sites prediction. *Protein Eng. Des. Sel.* 21 639–644. 10.1093/protein/gzn039 18753194 PMC2569006

[B60] Seigneuret M. (2006). Complete predicted three-dimensional structure of the facilitator transmembrane protein and hepatitis C virus receptor CD81: conserved and variable structural domains in the tetraspanin superfamily. *Biophys. J.* 90 212–227. 10.1529/biophysj.105.069666 16352525 PMC1367020

[B61] Seigneuret M. Delaguillaumie A. Lagaudrière-Gesbert C. Conjeaud H. (2001). Structure of the tetraspanin main extracellular domain. A partially conserved fold with a structurally variable domain insertion. *J. Biol. Chem.* 276 40055–40064. 10.1074/jbc.M105557200 11483611

[B62] Sezgin E. Levental I. Mayor S. Eggeling C. (2017). The mystery of membrane organization: composition, regulation and roles of lipid rafts. *Nat. Rev. Mol. Cell Biol.* 18 361–374. 10.1038/nrm.2017.16 28356571 PMC5500228

[B63] Shanmukhappa K. Kim J. K. Kapil S. (2007). Role of CD151, A tetraspanin, in porcine reproductive and respiratory syndrome virus infection. *Virol. J.* 4:62. 10.1186/1743-422x-4-62 17572908 PMC1906853

[B64] Sincock P. M. Fitter S. Parton R. G. Berndt M. C. Gamble J. R. Ashman L. K. (1999). PETA-3/CD151, a member of the transmembrane 4 superfamily, is localised to the plasma membrane and endocytic system of endothelial cells, associates with multiple integrins and modulates cell function. *J. Cell Sci.* 112(Pt. 6) 833–844. 10.1242/jcs.112.6.833 10036233

[B65] Singethan K. Schneider-Schaulies J. (2008). Tetraspanins: small transmembrane proteins with big impact on membrane microdomain structures. *Commun. Integr. Biol.* 1 11–13. 10.4161/cib.1.1.6406 19704780 PMC2633786

[B66] Stipp C. S. Kolesnikova T. V. Hemler M. E. (2003). Functional domains in tetraspanin proteins. *Trends Biochem. Sci.* 28 106–112. 10.1016/s0968-0004(02)00014-2 12575999

[B67] Thomas P. J. Qu B. H. Pedersen P. L. (1995). Defective protein folding as a basis of human disease. *Trends Biochem. Sci.* 20 456–459. 10.1016/s0968-0004(00)89100-8 8578588

[B68] Todres E. Nardi J. B. Robertson H. M. (2000). The tetraspanin superfamily in insects. *Insect Mol. Biol.* 9 581–590. 10.1046/j.1365-2583.2000.00222.x 11122467

[B69] van Deventer S. J. Dunlock V. E. van Spriel A. B. (2017). Molecular interactions shaping the tetraspanin web. *Biochem. Soc. Trans.* 45 741–750. 10.1042/bst20160284 28620035

[B70] van Spriel A. B. Figdor C. G. (2010). The role of tetraspanins in the pathogenesis of infectious diseases. *Microbes Infect.* 12 106–112. 10.1016/j.micinf.2009.11.001 19896556

[B71] Veneault-Fourrey C. Lambou K. Lebrun M. H. (2006). Fungal Pls1 tetraspanins as key factors of penetration into host plants: a role in re-establishing polarized growth in the appressorium? *FEMS Microbiol. Lett.* 256 179–184. 10.1111/j.1574-6968.2006.00128.x 16499604

[B72] Veneault-Fourrey C. Parisot D. Gourgues M. Laugé R. Lebrun M. H. Langin T. (2005). The tetraspanin gene ClPLS1 is essential for appressorium-mediated penetration of the fungal pathogen *Colletotrichum lindemuthianum*. *Fungal Genet. Biol.* 42 306–318. 10.1016/j.fgb.2005.01.009 15749050

[B73] Vinodhini J. Rajendran L. Raveendran M. Rajasree V. Karthikeyan G. (2020). Characterization of cucumber mosaic virus (CMV) subgroup IB infecting chilli in Tamil Nadu, India. *3 Biotech* 10:500. 10.1007/s13205-020-02492-y 33163319 PMC7604281

[B74] Wang F. Muto A. Van de Velde J. Neyt P. Himanen K. Vandepoele K. (2015). Functional analysis of the *Arabidopsis* TETRASPANIN gene family in plant growth and development. *Plant Physiol.* 169 2200–2214. 10.1104/pp.15.01310 26417009 PMC4634101

[B75] Wang F. Vandepoele K. Van Lijsebettens M. (2012). Tetraspanin genes in plants. *Plant Sci.* 190 9–15. 10.1016/j.plantsci.2012.03.005 22608515

[B76] Yánez-Mó M. Mittelbrunn M. Sánchez-Madrid F. (2001). Tetraspanins and intercellular interactions. *Microcirculation* 8 153–168. 10.1038/sj/mn/7800076 11498779

[B77] Yang X. Claas C. Kraeft S. K. Chen L. B. Wang Z. Kreidberg J. A. (2002). Palmitoylation of tetraspanin proteins: modulation of CD151 lateral interactions, subcellular distribution, and integrin-dependent cell morphology. *Mol. Biol. Cell* 13 767–781. 10.1091/mbc.01-05-0275 11907260 PMC99597

[B78] Yang X. Kovalenko O. V. Tang W. Claas C. Stipp C. S. Hemler M. E. (2004). Palmitoylation supports assembly and function of integrin-tetraspanin complexes. *J. Cell Biol.* 167 1231–1240. 10.1083/jcb.200404100 15611341 PMC2172609

[B79] Zhou F. Xue Y. Yao X. Xu Y. (2006). CSS-Palm: palmitoylation site prediction with a clustering and scoring strategy (CSS). *Bioinformatics* 22 894–896. 10.1093/bioinformatics/btl013 16434441

[B80] Zöller M. (2009). Tetraspanins: push and pull in suppressing and promoting metastasis. *Nat. Rev. Cancer* 9 40–55. 10.1038/nrc2543 19078974

