# Supplementary figures and images for: Arabidopsis Tetraspanins Facilitate Virus Infection via Membrane-Recognition GCCK/RP Motif and Cysteine Residues
Source: Front Plant Sci. 2022 Mar 3;13:805633. doi: 10.3389/fpls.2022.805633 (PMC8927881; doi:10.3389/fpls.2022.805633)

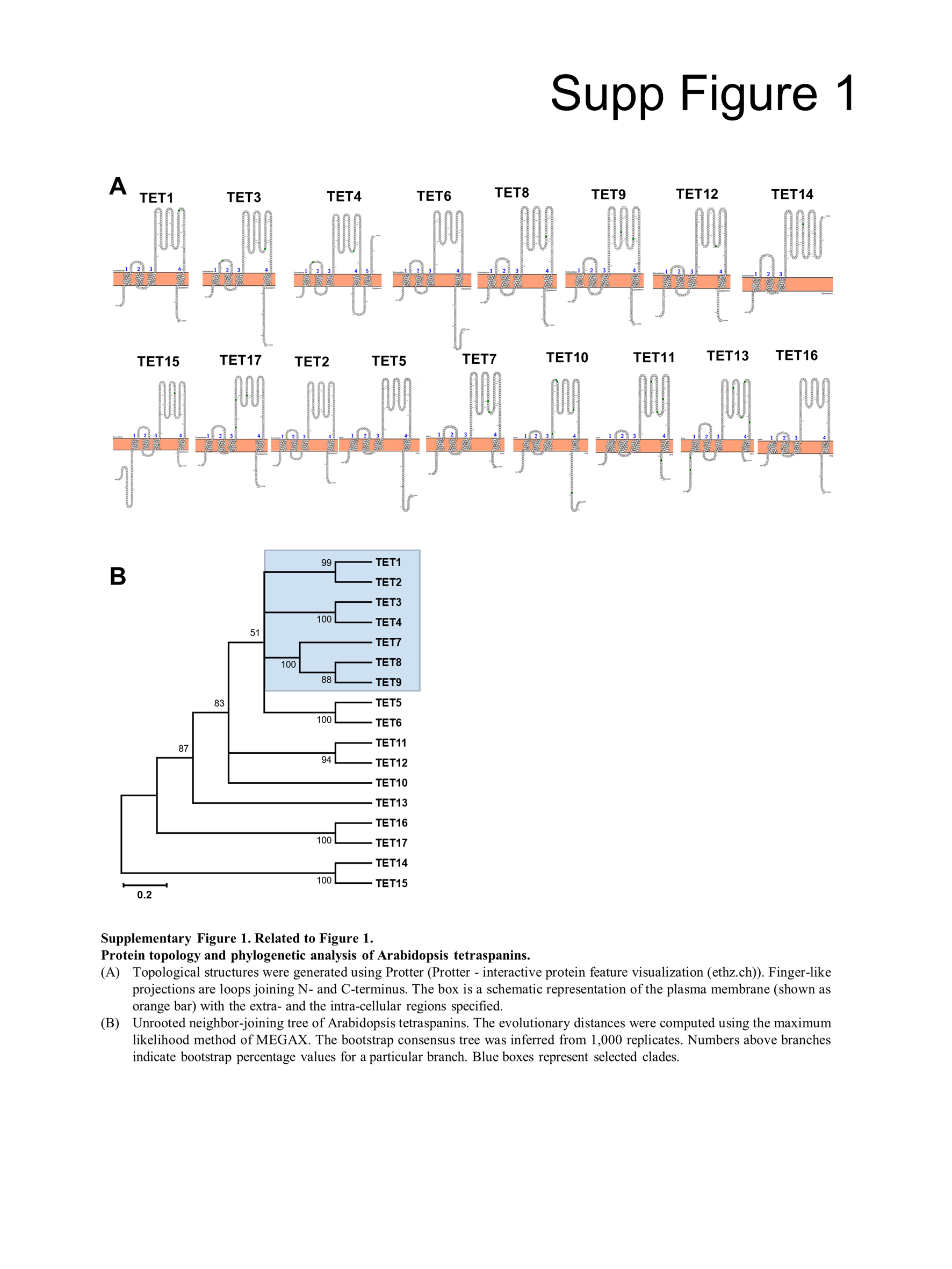

Supplement: Supplementary file 3 [file Image_1.TIF]

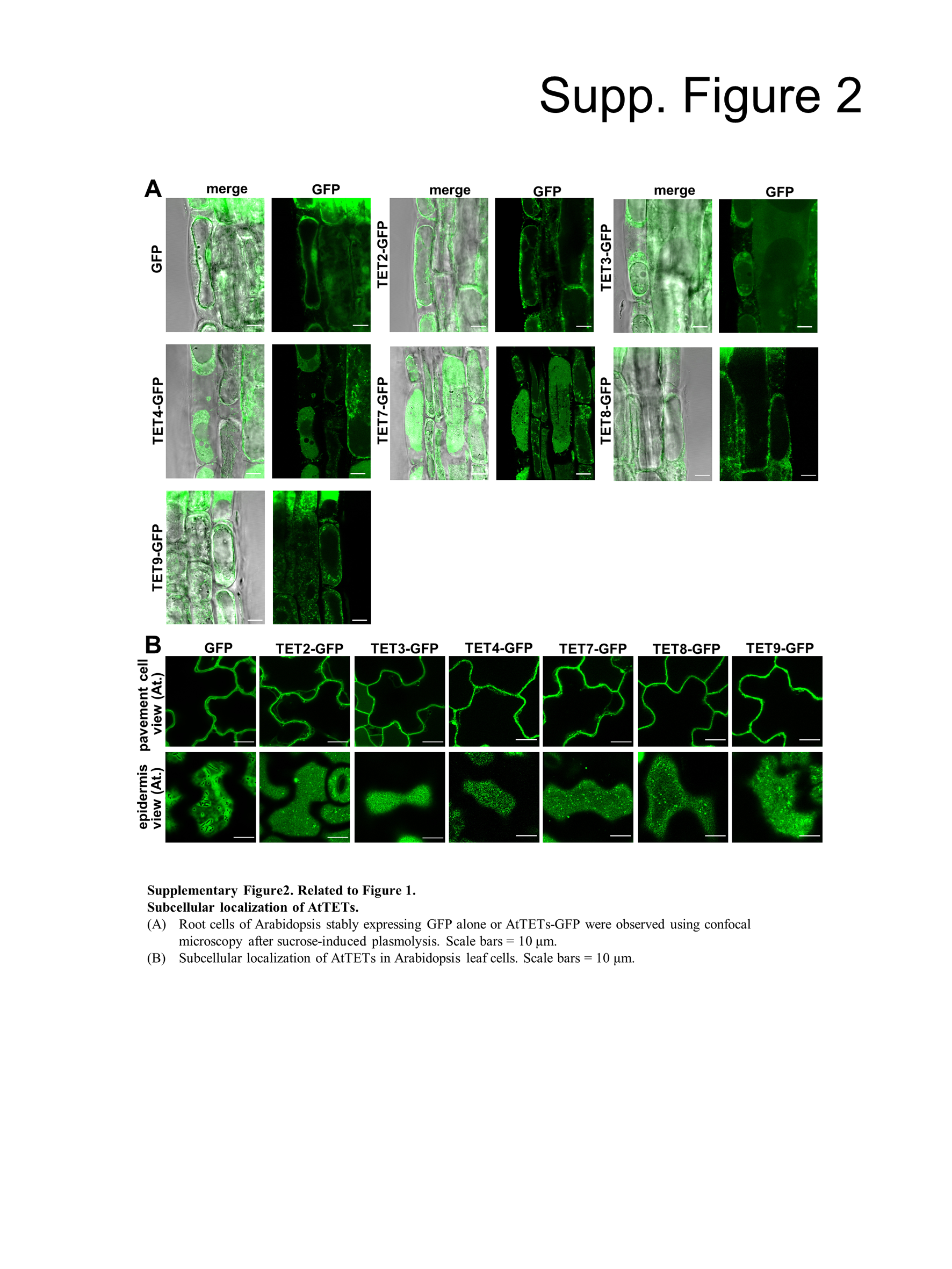

Supplement: Supplementary file 4 [file Image_2.TIF]

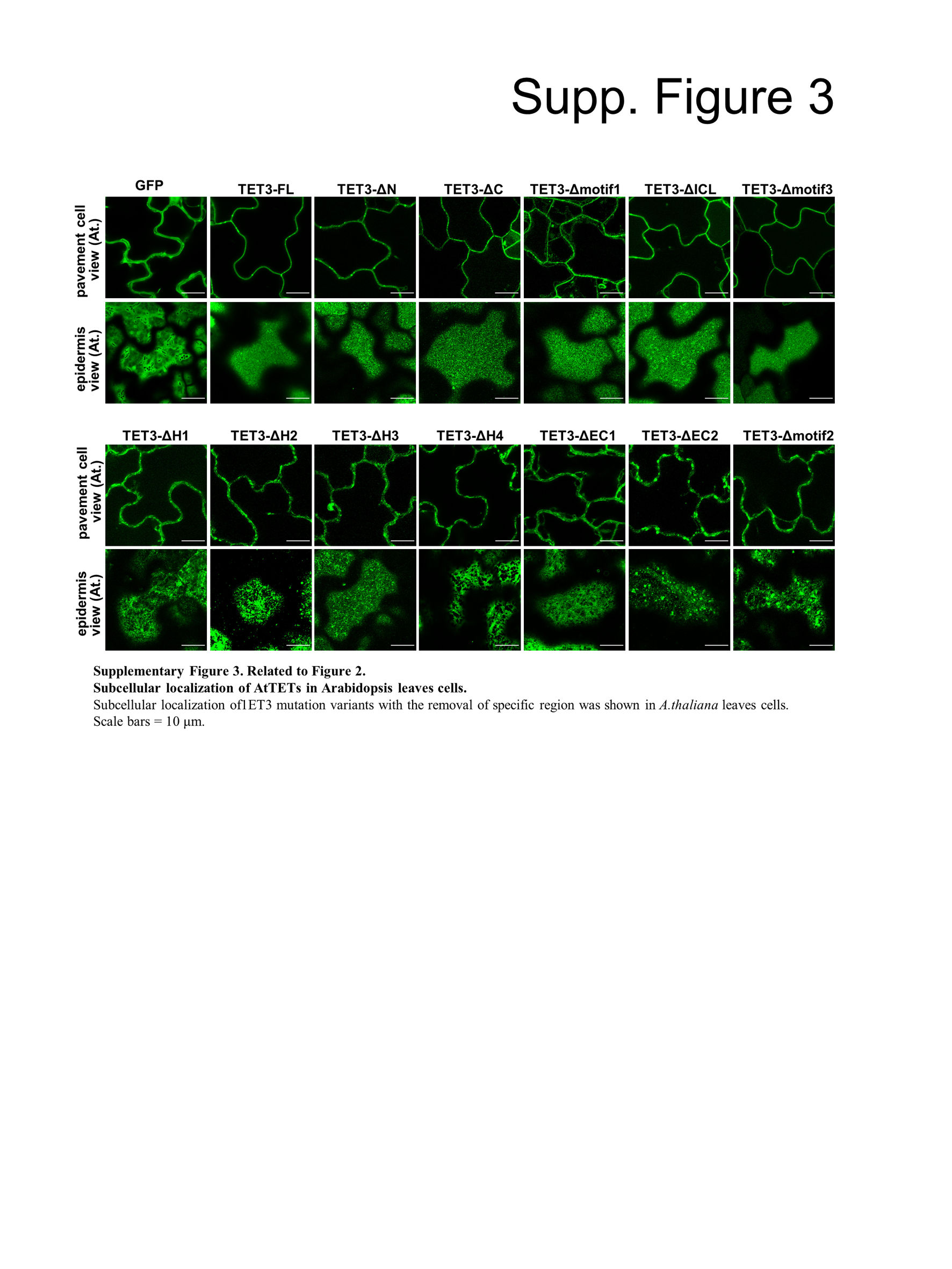

Supplement: Supplementary file 5 [file Image_3.TIF]

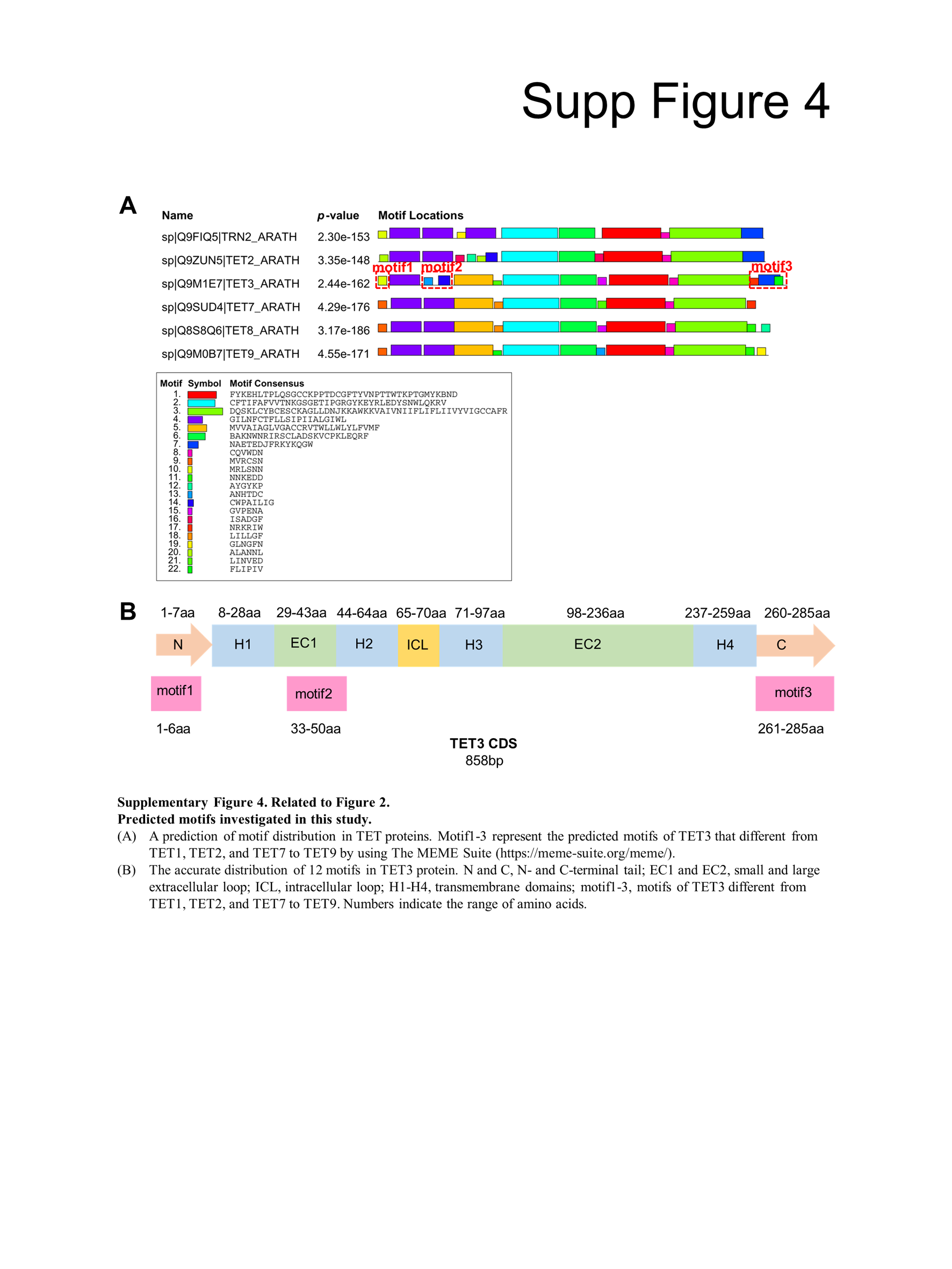

Supplement: Supplementary file 6 [file Image_4.TIF]

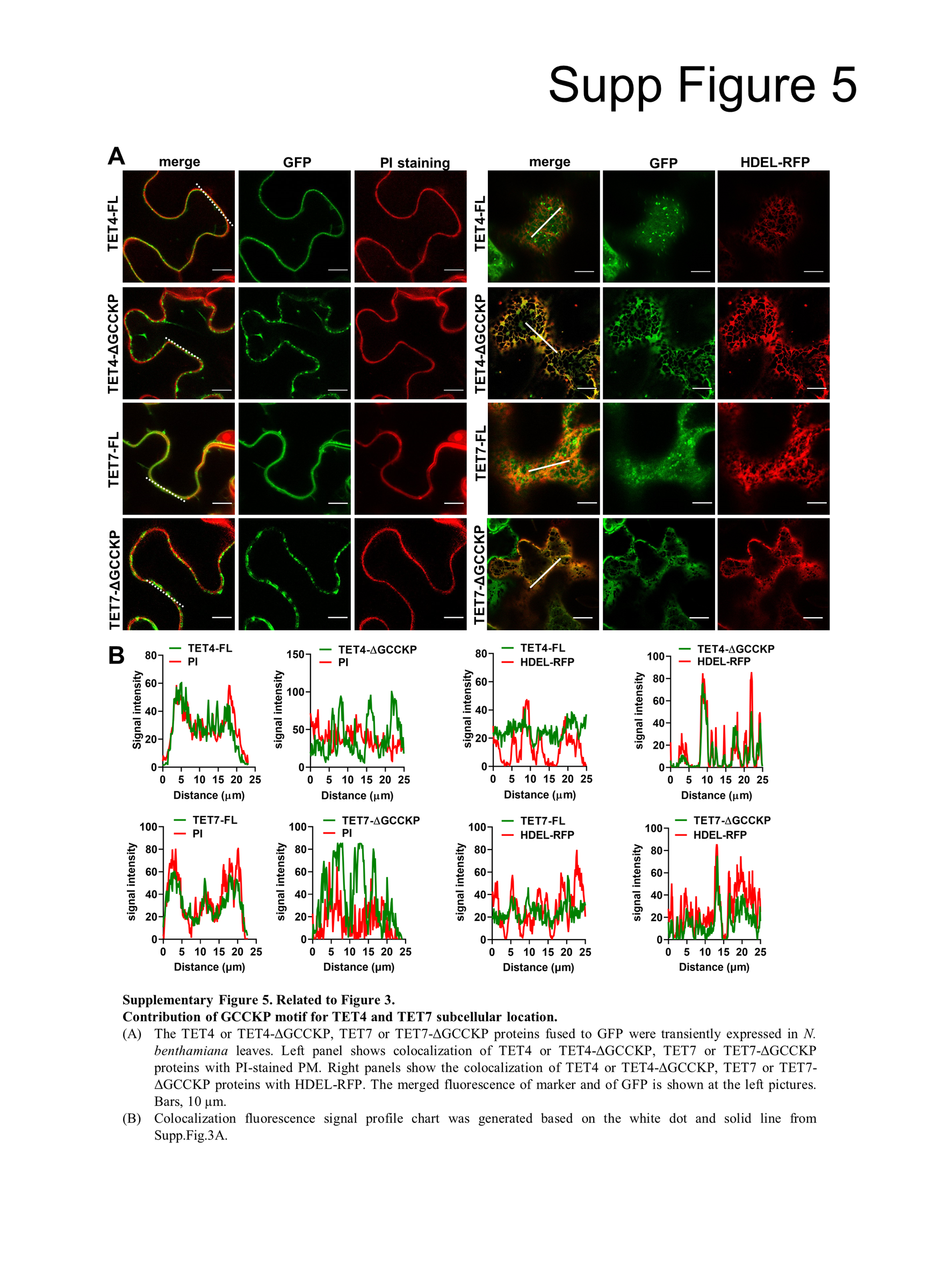

Supplement: Supplementary file 7 [file Image_5.TIF]

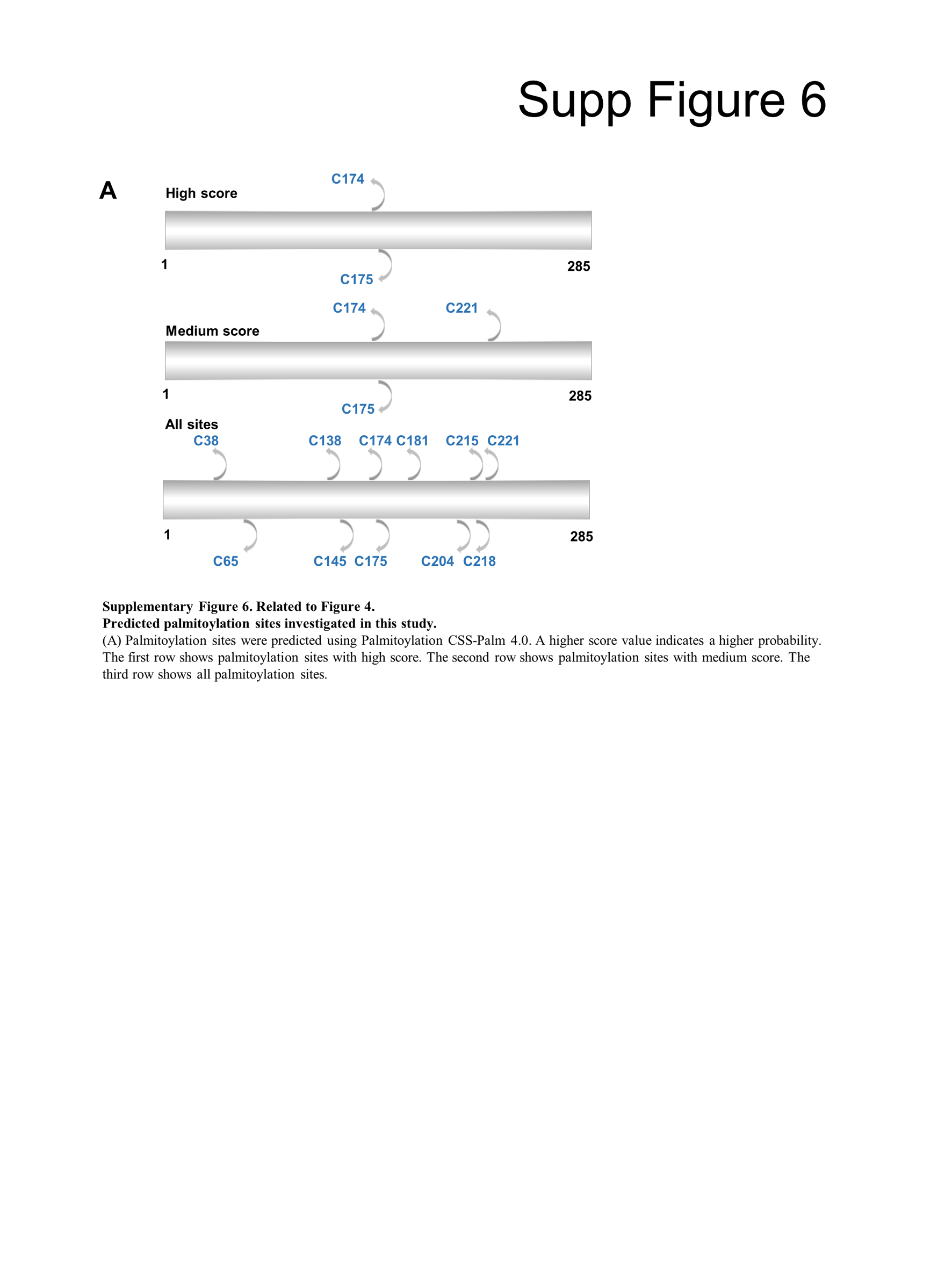

Supplement: Supplementary file 8 [file Image_6.TIF]

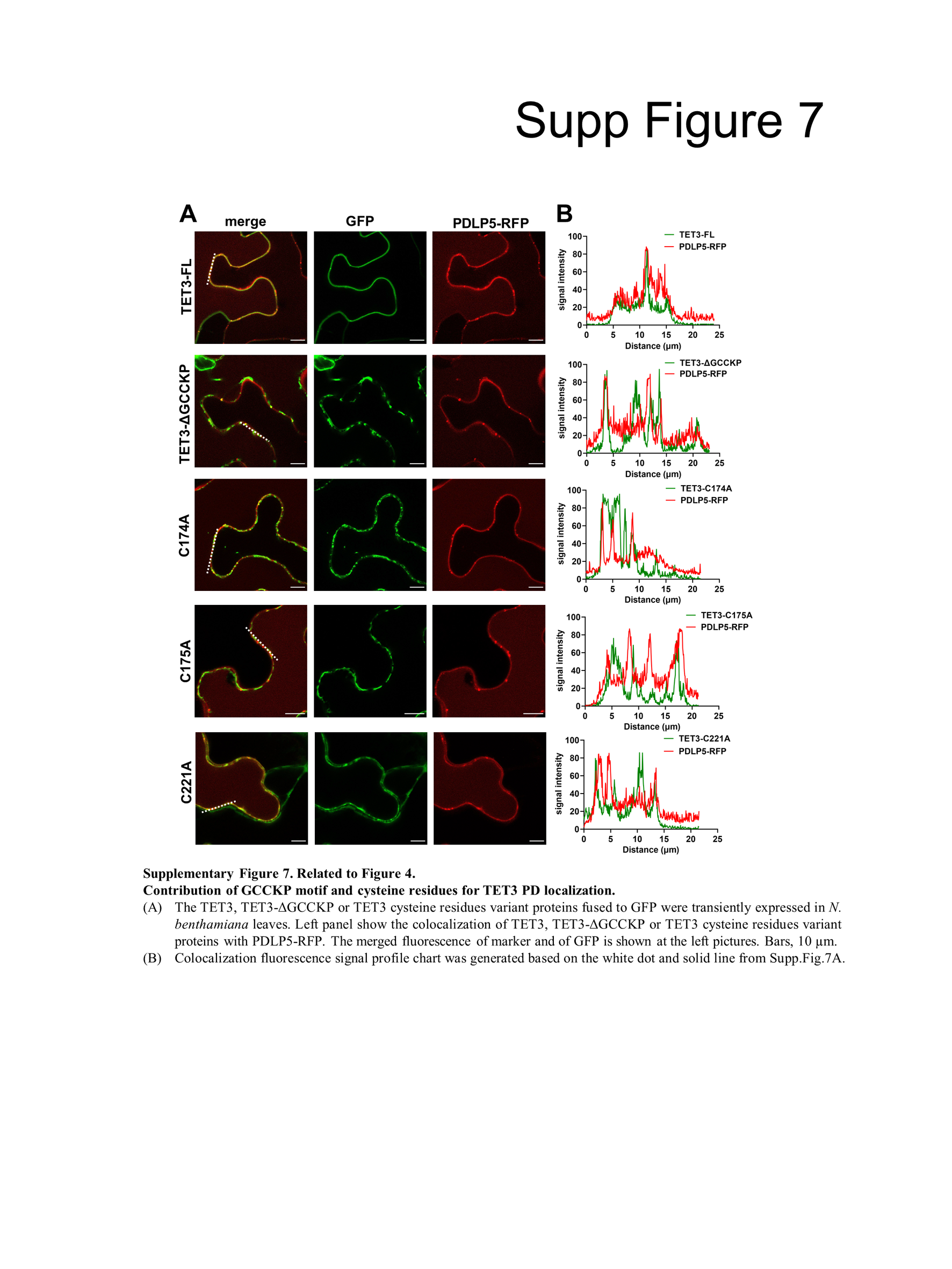

Supplement: Supplementary file 9 [file Image_7.TIF]

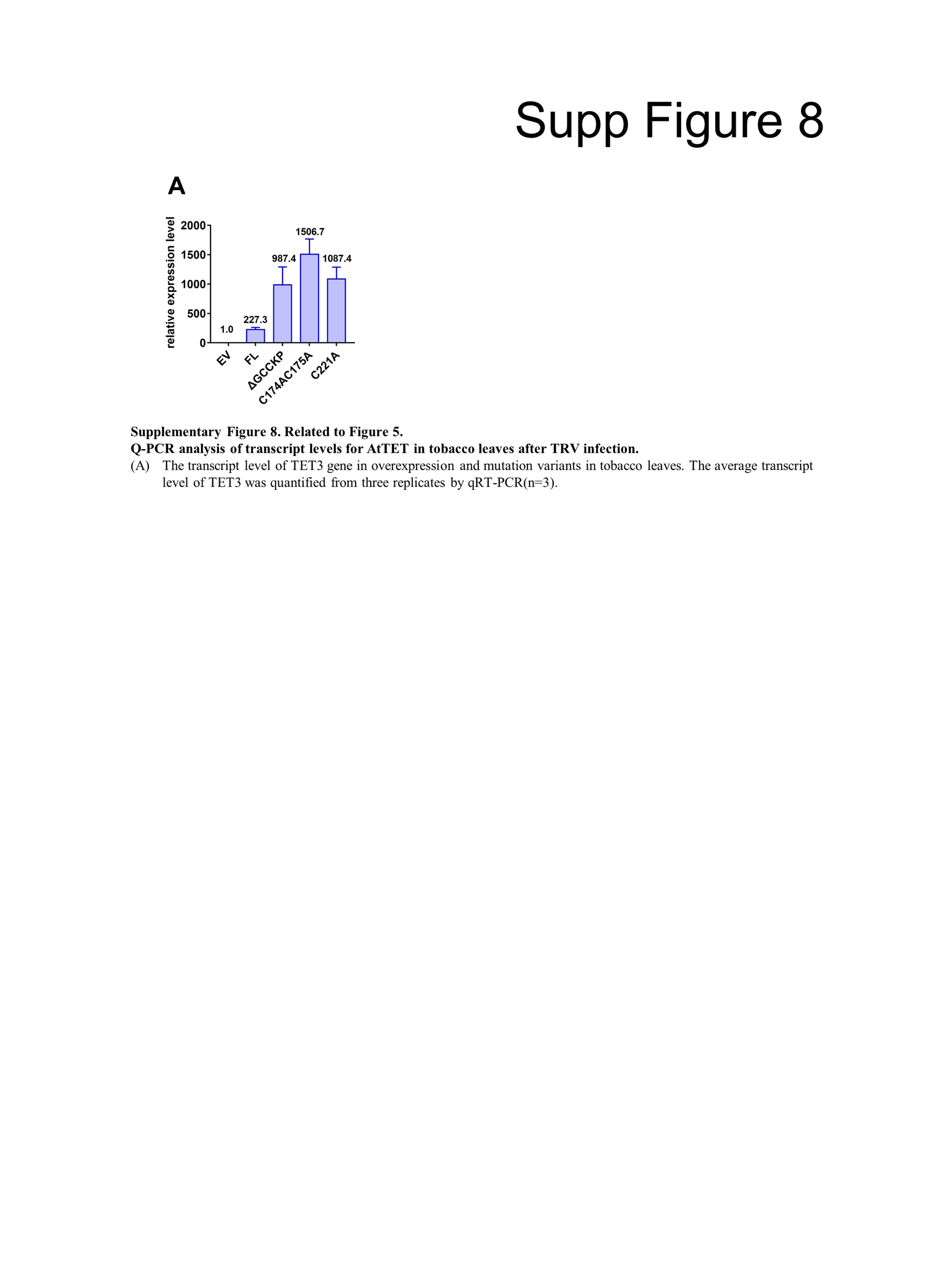

Supplement: Supplementary file 10 [file Image_8.TIF]
